# Molecular systematics of the Labeonini inhabiting the karst regions in southwest China (Teleostei, Cypriniformes)

**DOI:** 10.3897/zookeys.612.9085

**Published:** 2016-08-23

**Authors:** Lan-Ping Zheng, Xiao-Yong Chen, Jun-Xing Yang

**Affiliations:** 1 State Key Laboratory of Genetic Resources and Evolution, Kunming Institute of Zoology, the Chinese Academy of Sciences, 32 Jiaochang Donglu, Kunming 650223, Yunnan, China; 2 Southeast Asia Biodiversity Research Institute, the Chinese Academy of Sciences, (CAS-SEABRI), Nay Pyi Taw 05282, Yezin, Myanmar

**Keywords:** China, karst regions, Labeonini, molecular systematics, taxonomic revision

## Abstract

The major phylogenetic pattern of the cyprinid tribe Labeonini has been revealed by previous molecular studies; however, the relationships within a clade that mainly inhabits the karst regions, which we refer to as the “karst group”, in southwest China remain unresolved due to the low taxon sampling. This group includes more than 50% of the genera and species of Labeonini in China. Moreover, more than 90% of the genera of this group are endemic to China. In addition, some new genera and species of Labeonini have been discovered from these karst regions, but their taxonomic validity and phylogenetic position have not been examined. In this contribution, partial sequences of four nuclear (exon 3 of recombination activating protein 1, rhodopsin, early growth response protein 2B gene and interphotoreceptor retinoid binding protein gene) and three mitochondrial genes (cytochrome *b*, cytochrome oxidase subunit I and 16S ribosomal RNA) from 36 ingroup taxa and 25 outgroup taxa were analyzed to provide a hypothesis of the phylogenetic relationships within the labeonins of the karst regions in China. We propose that the monophyly of *Parasinilabeo*, *Ptychidio*, *Rectoris* and *Semilabeo* are supported. A new genus, *Prolixicheilus*, is erected for *Pseudogyrinocheilus longisulcus. Cophecheilus bamen* is the sister to *Prolixicheilus longisulcus*. *Ptychidio*, *Pseudocrossocheilus*, *Semilabeo*, *Rectoris* and *Stenorynchoacrum* are closely related with high support values. *Sinocrossocheilus*, *Pseudogyrinocheilus*, *Paraqianlabeo*, *Hongshuia*, *Discogobio* and *Discocheilus* form a clade together with high support. Considering molecular results and morphological differences, *Parasinilabeo longicorpus* and *Ptychidio macrops* might be the synonyms of *Parasinilabeo assimilis* and *Ptychidio jordani* respectively. Comprehensive taxonomic revisions of the two genera *Parasinilabeo* and *Ptychidio* may be necessary.

## Introduction

Fishes of the tribe Labeonini (Cypriniformes: Cyprinidae) are adapted to riverine environments. Labeonini used here is equal to Labeoninae in Zheng et al. (2010, 2012). They have evolved a diverse mouth morphology. The diversity of these morphological characters has been used to identify genera and generate hypotheses of phylogeny (Zhang 1994, 1998a, b, 2005; Zhang et al. 2000). Therefore, the species of Labeonini with similar oral morphology were thought to be closely related by these morphological studies. As the development of molecular techniques has advanced, the results of previous morphological phylogenetic studies have been challenged. Recent molecular studies demonstrated a different phylogenetic pattern of Labeonini from that derived from morphology. Species with similar morphology were not closely related to each other in the molecular studies (Yang et al. 2010; 2012; Zheng et al. 2010; 2012). The relationships within Labeonini were basically consistent in the aforementioned molecular studies. However, the relationships within the terminal clade of Labeonini were unresolved due to a low taxon sampling. This terminal clade is equal to the Clade F in Zheng et al. (2010). This clade mainly inhabits the karst regions in China’s southwestern provinces: Yunnan, Guizhou and Guangxi, which is characterized by a mass of underground rivers and caves. Therefore, we define it as the karst group herein. The karst group included 52 species within 14 genera, accounting for 57% of the species and 55% of all the genera of the Labeonini in China. Moreover, more than 90% of the genera of this group are endemic to China (Table 1). Yang et al. (2010) refer to a single species in each of 7 genera inhabiting the karst regions, and Zheng et al. (2010, 2012) to 23 species distributed over 12 genera. Yang et al. (2012) dealt with the same genera as Zheng et al. (2010, 2012) adding three more species. It is obvious that previous studies suffered from low taxon sampling, leading to yet unresolved specific phylogenetic relationships within the karst group.

**Table 1.** List of genera and species of Labeonini inhabiting the karst regions of China.

Genus name	Number of species	Distribution
*Discocheilus* Zhang, 1997	2	China
*Discogobio* Lin, 1931	16	China (13), Vietnam (3)
*Hongshuia* Zhang, Qing & Lan, 2008	3	China
*Parasinilabeo* Wu, 1939	6	China
*Pseudocrossocheilus* Zhang & Chen, 1997	6	China
*Pseudogyrinocheilus* Fang, 1933	2	China
*Ptychidio* Myers, 1930	3	China
*Qianlabeo* Zhang & Chen, 2004	1	China
*Rectoris* Lin, 1935	5	China
*Semilabeo* Peters, 1881	2	China
*Sinocrossocheilus* Wu, 1977	2	China
*Stenorynchoacrum* Huang, Yang & Chen, 2014	1	China
*Cophecheilus* Zhu, Zhang, Zhang & Han, 2011	2	China
*Paraqianlabeo* Zhao, Sullivan, Zhang & Peng, 2014	1	China
**Sum**	52	

Several new genera, such as *Qianlabeo* Zhang & Chen, 2004, *Hongshuia* Zhang, Qing & Lan, 2008, *Cophecheilus* Zhu, Zhang, Zhang & Han, ﻿2011, *Sinigarra* Zhang & Zhou, 2012, *Stenorynchoacrum* Huang, Yang & Chen, 2014, and *Paraqianlabeo* Zhao, Sullivan, Zhang & Peng, 2014, and some new species, such as *Parasinilabeo longicorpus* Zhang, 2000, *Parasinilabeo longibarbus* Zhu, Lan & Zhang, 2006, *Parasinilabeo longiventralis* Huang, Chen & Yang, 2007, and *Pseudogyrinocheilus longisulcus* Zheng, Chen & Yang, 2010, have been described since 2000. All descriptions were based on morphological characters, in particular on the structural morphology of the mouth. These recently described genera and species are all distributed in karst regions in southwest China. The phylogenetic positions of some new genera and species have not yet been examined. Studies of Labeonini indicated that these morphological characters evolved homoplastically (Zheng et al. 2012). Therefore, the phylogenetic positions of the new genera and species need to be further examined.

This contribution reconstructs the phylogenetic tree based on extensive sampling and multiple molecular markers in order to demonstrate the phylogenetic relationships of the karst group.

## Materials and methods

### Sample collection

At least two specimens of each species were sequenced and analyzed, and all the specimens of the same species shared a common haplotype or clustered into a lineage. Each species is represented by one specimen (two for *Parasinilabeo longicorpus*). A total of 37 specimens representing 36 species and 13 genera of the karst group were used in this work. Eleven species of Cyprininae were selected as distant outgroups and 14 species of Labeonini were selected as hierarchical outgroups, following Mayden et al. (2009) and Zheng et al. (2010). Species identification and collection localities are given in Suppl. material 1. All voucher specimens sequenced for use in this study are deposited in the Kunming Institute of Zoology, the Chinese Academy of Sciences.

### DNA extraction, PCR amplification and sequencing

The genomic DNA was extracted from fin clips preserved in 95% ethanol. Three mitochondrial genes (cytochrome *b*, cytochrome oxidase subunit I, and 16S ribosomal RNA) and four nuclear genes (exon 3 of recombination activating protein 1 (RAG1), Rhodopsin (RH), early growth response protein 2B gene (EGR2B) and interphotoreceptor retinoid binding protein gene (IRBP)) have been used in this study. The primers for mitochondrial genes for PCR amplification have been given in Zheng et al. (2010), and nuclear genes followed Chen et al. (2008). Sequencing was performed directly using the corresponding PCR primers. PCR products were purified via spin columns. Purified PCR products were sequenced in both forward and reverse directions using the sequencing services of BigDye Terminator v3.1 on an ABI PRISM 3730 following the manufacturer’s instructions. All sequence accession numbers are given in Suppl. material 1.

### Statistical analyses

Sequences were aligned using ClustalX v1.83 (Thompson et al. 1997) and manually checked for inconsistencies. To test for the possible saturation of substitution types, the number of transitions (Ti) and transversions (Tv) versus the F84 distance were plotted for our sequences in DAMBE (Xia and Lemey 2009). The base compositional bias using a chi-square test with the BaseFreq function implemented in PAUP* 4.0b 10 (Swofford 2002).

### Phylogenetic analyses

Phylogeny reconstruction was carried out with Bayesian (BI) and maximum likelihood (ML) approaches. The most appropriate evolutionary model was selected by Modeltest v3.7 (Posada and Crandall 1998) for BI and ML using Akaike information criterion (AIC, Nylander et al. 2004) before phylogenetic analyses. Bayesian analysis was conducted using MrBayes 3.1.2 (Huelsenbeck and Ronquist 2001). Four chains (three hot, one cold) were run for 10,000,000 generations, sampling trees every 100 generations and with the first 25,000 generations discarded as burn-in. Convergence was confirmed by ascertaining that the average standard deviation of split frequencies was below 0.01. Six data partitioning strategies were adopted in the Bayesian analysis on the combined data set, with the number of data partitions ranging from 1 (all genes evolve under a single evolutionary model) to 11 (partitions for each of the 2 protein coding genes plus 5 separate partition for 16S rRNA, RAG1, RH, EGR2B and IRBP) (Table 2). The program PartitionFinder was used to select the partition scheme and evolutionary models for our sequences (Lanfear et al. 2012). Partitioning strategies were compared by Bayes factors, which represent the ratio of the harmonic mean likelihoods of the two analyses being tested in MrBayes 3.1.2. For each run, the harmonic mean likelihoods were calculated using the ‘sump’ command. A value greater than 5 for ln Bayes factor was considered as strong evidence against the alternative topology tested (Kass and Raftery 1995). The optimal partition selected by Bayes factor was used in Maximum Likelihood analysis. Partitioned ML analysis employing separate models was performed using GARLI 2.0 (Zwickl 2006) with model parameters optimized during the run. Nodal support was assessed by 1000 bootstrap replicates and then the resulting bootstrap trees were imported into PAUP* 4.0b 10 (Swofford 2002) to obtain the bootstrap values and a majority-rule consensus topology.

**Table 2.** Partitioning strategies used in this study.

#	Partition strategy	Partition identity
P1	All data combined	COI+Cyt *b*+16S+RAG1+RH+EGR2B+IRBP
P5	By mitochondrial and nuclear genes	COI+Cyt *b*+16S; RAG1; RH; EGR2B; IRBP
P6	Based on the analysis of our combined dataset using PartitionFinder	Cyt *b*1+RAG1; Cyt *b* 2+COI 3+16SrRNA +EGR2B; Cyt *b* 3; COI 1+RH; COI 2; IRBP
P7	By gene	COI; Cyt *b*; 16S; RAG1; RH; EGR2B; IRBP
P9	By separating codon positions 1 & 2 and codon position 3 of protein-coding gene, non-coding mitochondrial gene and nuclear gene	COI 1 2; COI 3+Cyt *b* 1 2; Cyt *b* 3; 16S; RAG1; RH; EGR2B; IRBP
P11	By codon position of protein-coding mitochondrial gene, non-coding mitochondrial gene and nuclear gene	COI 1; COI 2; COI 3; Cyt *b* 1; Cyt *b* 2;Cyt *b* 3; 16S; RAG1; RH; EGR2B; IRBP

BI and ML tree were tested using the Shimodaira–Hasegawa (SH) test (Shimodaira and Hasegawa 1999) in PAUP* 4.0b 10, using 1000 bootstrap replicates with RELL optimization. The RELL approximation is used to avoid the re-estimation of the parameters in the bootstrap replicates (Buckley et al. 2001).

## Results

### Sequence analyses

A total of 402 nucleotide sequences were used in this study, of which 106 sequences were obtained from this study and 296 downloaded from the GenBank. No signal of saturation was observed among sequences (Suppl. material 2). A total of 6600 bp nucleotides were used in the analyses, including 837 bp of COI, 1098 bp of Cyt *b*, 1151 bp of 16S rRNA, 1465 bp of RAG1, 488 bp of RH, 751 bp of EGR2B and 810 bp of IRBP. Mean base composition of the combined dataset is as follows: A, 0.2821; C, 0.2844; G, 0.1913, and T, 0.2422. No significant compositional biases existed in either ingroup or outgroup taxa (P=1.00>0.05). Nucleotide substitution models selected by AIC under different partition models are presented in Table 3. The mean ln likelihood (ln L) and Bayes factor comparisons are presented in Table 4. The partitioned scheme separated by codon positions 1 and 2 and codon position 3 of protein-coding gene, non-coding mitochondrial and nuclear gene (P9) was selected as the best-fit partition scheme.

**Table 3.** Nucleotide substitution models selected by AIC under different partition models.

Gene	Model
COI	GTR+I+G
COI 1st position	GTR+I+G
COI 2nd position	HKY
COI 1st and 2nd position	GTR+I+G
COI 3rd position	GTR+I+G
Cyt *b*	GTR+I+G
Cyt *b* 1st position	GTR+I+G
Cyt *b* 2nd position	GTR+I+G
Cyt *b* 1st and 2nd position	TIM+I+G
Cyt *b* 3rd position	TIM+G
16S	GTR+I+G
RAG1	SYM+I+G
RH	K81uf+I+G
EGR2B	TrN+I+G
IRBP	TrNef+I+G
models selected by partitionfinder	
Cyt *b*1st position +RAG1	SYM+I+G
Cyt *b* 2nd+COI 3 rd position +16SrRNA +EGR2B	GTR+I+G
Cyt *b* 3rd position	GTR+G
COI 1st position +RH	TIM+I+G
COI 2nd position	HKY+I
IRBP	TrNef+I+G

**Table 4.** Comparison of likehood scores after different partitioning strategies and estimation of Bayes factors. Bayes factors are calculated as 2(Px-PY).

Partition	-lnL	P5	P7	P11	P1	P6
P9	86281.43	711.74	1527.96	7810.76	8932.12	16455.34
P5	86637.30		816.22	7099.02	8220.38	15743.6
P7	87045.41			6282.8	7404.16	14927.38
P11	90186.81				1121.36	8644.58
P1	90747.49					7523.22
P6	94509.10					

### Phylogenetic analyses

**Figure 1.**
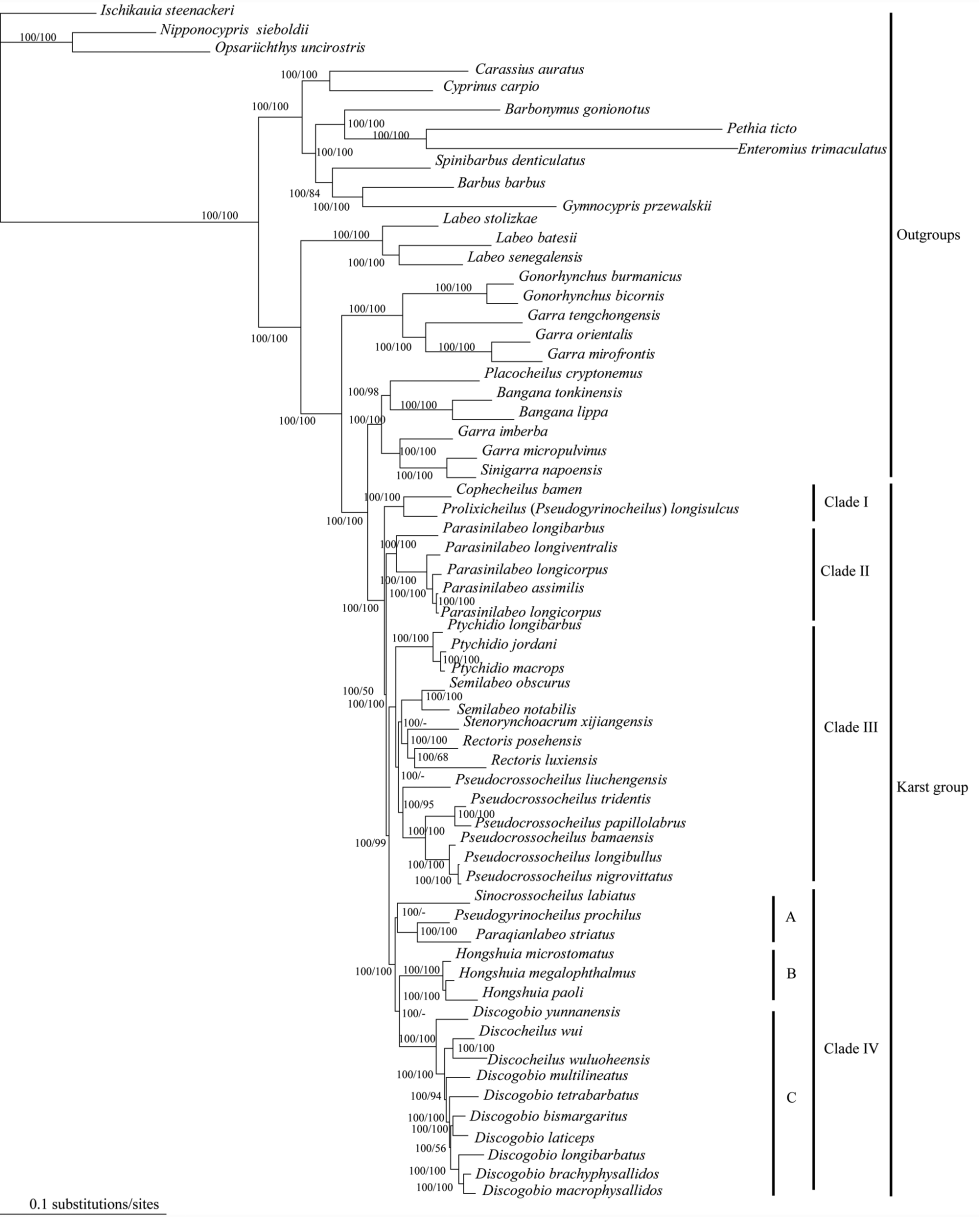
Phylogenetic tree derived from a partitioned Maximum Likelihood analysis of the combined data set. The nodal numbers are ML bootstrap values and Bayesian posterior probabilities, respectively. Only values above 50% are given.

The SH test did not reject any hypotheses of BI or ML (P>0.05). Relationships of all taxa derived from partitioned ML and Bayesian analyses of sequences were nearly identical. Thus, the ML tree is presented here together with the nodal support values generated by ML bootstrap analysis and Bayesian posterior probabilities (BPPs), respectively (Fig. 1). All phylogenetic analyses show that the group of the labeonins in the karst regions of China is divided into four lineages (Fig. 1).

1) *Pseudogyrinocheilus longisulcus* Zheng, Chen & Yang, 2010 forms the sister taxon to *Cophecheilus bamen* Zhu, Zhang, Zhang & Han, 2011, and together they form the first lineage Clade I.

2) The monophyly of *Parasinilabeo* is not rejected and all the species of *Parasinilabeo* form the second lineage Clade II.

3) The species of *Ptychidio*, *Pseudocrossocheilus*, *Semilabeo*, *Rectoris* and *Stenorynchoacrum* form a monphyletic group, and the third lineage in our study. The monophyly of *Ptychidio*, *Pseudocrossocheilus*,﻿ *Rectoris* and *Semilabeo* are not rejected by all analyses, while *Stenorynchoacrum xijiangensis* Huang, Yang & Chen, 2014 forms the sister taxon to *Rectoris*.

4) *Sinocrossocheilus*, *Pseudogyrinocheilus*, *Paraqianlabeo*, *Hongshuia*, *Discogobio*, and *Discocheilus* form the forth lineage (Clade IV), which can be further divided into three subclades (Clade IV A-C). Within Clade IV, *Sinocrossocheilus*, *Pseudogyrinocheilus* and *Paraqianlabeo* form Clade IV A. The monophyly of *Hongshuia* is supported and all the species of *Hongshuia* form Clade IV B. *Discocheilus* and *Discogobio* form Clade IV C together.

### Taxonomic revision

*Pseudogyrinocheilus longisulcus* was described as a new species of *Pseudogyrinocheilus* because it shares similar mouth morphology with *Pseudogyrinocheilus prochilus* (Sauvage & Dabry de Thiersant, 1874) (Zheng et al. 2010). However, the molecular results show that *Pseudogyrinocheilus longisulcus* and *Pseudogyrinocheilus prochilus* are located into two distant lineages. It is indicated that the oral morphological character evolved convergently and the allocation of this species need to be revised. Therefore, we erect a new genus for *Pseudogyrinocheilus longisulcus*.

#### Prolixicheilus gen. n.

Taxon classificationAnimaliaCypriniformesCyprinidae

http://zoobank.org/3CB3F6C1-5F77-403B-85D5-60D6F14FCEEA

##### Type species.

**Figure 2.**
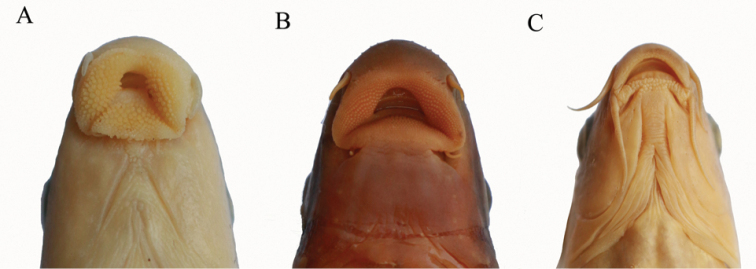
Ventral view of the mouth morphology. **A** *Pseudogyrinocheilus longisulcus* **B** *Pseudogyrinocheilus prochilus* **C** *Cophecheilus bamen*.

*Pseudogyrinocheilus longisulcus* Zheng, ﻿Chen & Yang,﻿ 2010 (Fig. 2A).

##### Etymology.

From the Latin adjective *prolixus*, meaning broad, stretched far out, and the Greek noun *cheilos* meaning lip, an allusion to the broad lips of the type species. Gender masculine.

##### Diagnosis.

*Prolixicheilus* can be distinguished from all other genera of labeonins by its peculiar morphology: papillate rostral fold and lower lip, evaginating and triangular; rostral fold pendulous, expanded ventrally, posterior margin non-fimbriate; lower lip with a straight posterior margin; upper lip vestigial; postlabial grooves prolonged, and extended anteromedially close to anterior end of middle lower lip, but not meeting with its counterpart; posterior margin of lower lip free; lateral-line scales 40–42; a longitudinal dark stripe along lateral line on flank; body laterally compressed.

##### Remarks.

*Prolixicheilus* can be easily distinguished from *Pseudogyrinocheilus* by the following combination of characteristics: postlabial grooves prolonged, and extended anteromedially close to anterior end of middle lower lip, but not meeting with its counterpart (only restricted at corners of mouth); posterior margin of lower lip free (vs. connected with chin); lateral-line scales 40–42 (vs. 45–49); a longitudinal dark stripe along lateral line on flank (vs. absent); body laterally compressed (vs. cylindrical). In addition, although *Pseudogyrinocheilus longisulcus* and *Cophecheilus bamen* are genetically closely related, *Pseudogyrinocheilus longisulcus* is readily distinguished from the species of *Cophecheilus* by the following combination of characteristics: rostral fold and lower lip evaginating (vs. not evaginating); rostral fold pendulous, expanded ventrally (vs. not pendulous, rostral cap with a shallow, arched, subdistal depression extending nearly the full length of its ventral edge); rostral fold and lower lip broad and fully papillated (vs. only margin papillated); posterior margin of lower lip free (vs. connected with chin); lateral-line scales 40–42 (vs. 43–48).

##### Distribution.

*Prolixicheilus longisulcus* has been only recorded in an unnamed stream in Lutong Village, Jingxi Co., Guangxi. The stream belongs to Zuojiang River, a tributary of Pearl River.

## Discussion

### Phylogenetic relationships

Previous studies on the molecular systematics of Labeonini included low taxonomic sampling of species from the karst regions of China. This and the close genetic relationships within this group are reflected by relatively low node values (Yang et al. 2010, 2012; Zheng et al. 2010, 2012) thereby indicating that the relationships within this group of labeonins have not been resolved satisfactorily. Moreover, the phylogenetic position of *Parasinilabeo*, *Ptychidio*, *Semilabeo*, *Rectoris* and *Stenorynchoacrum* were in a state of flux. Our results are very different from that of previous studies mentioned above and this group of Labeonini can be further divided into four clades with strong support. The monophyly of *Parasinilabeo*, *Ptychidio*, *Rectoris* and *Semilabeo* are firstly verified in this study, and the phylogenetic position of the genera listed above reach a definite conclusion.

In previous studied of the Labeonini, mouth morphology was used as an important character for taxonomy and phylogeny. Zhang (1994, 1998a) thought *Pseudogyrinocheilus*, *Semilabeo* and *Discocheilus* formed a monophyletic group, and that *Parasinilabeo* was closely related to both *Pseudogyrinocheilus* and *Semilabeo*. He also considered that *Sinocrossocheilus* was closely related to both *Pseudocrossocheilus* and *Rectoris* because these species share the same mouth structures, and he suggested that the four disc-bearing genera *Discocheilus*, *Discogobio*, *Garra* and *Placocheilus* formed a monophyletic group (Zhang 1998b, 2005).

The molecular results presented here show that species with similar morphological characters do not cluster in the phylogenetic tree. For example, *Ptychidio*, *Semilabeo*, *Stenorynchoacrum*, *Rectoris* and *Pseudocrossocheilus* form clade III. However, the margin of rostral fold of *Pseudocrossocheilus*, *Rectoris* and *Ptychidio* is crenulated with a deeply indented distal margin, and that of *Semilabeo* and *Stenorynchoacrum* is smooth or only with a median incision. *Pseudogyrinocheilus prochilus* does not have an oral disc on the lower lip, but form clade IV with disc-bearing species or species with a disc similar structure on the lower lip. *Paraqianlabeo striatus* Zhao, Sullivan, Zhang & Peng, 2014 has a well-developed upper lip, but other species included in the same clade have not. This indicates that the phylogenetic relationships of these species cannot be inferred by a few oral morphological characters.

### Phylogenetic positions of recently described genera

*Hongshuia*, *Cophecheilus*, *Sinigarra*, *Stenorynchoacrum* and *Paraqianlabeo* were described recently (Zhang et al. 2008; Zhu et al. 2011; Zhang and Zhou 2012; Huang et al. 2014; Zhao et al. 2014). The phylogenetic positions of *Cophecheilus* and *Sinigarra* have never been verified. Zhu et al. (2011) thought *Cophecheilus* is likely located in the basal position of the Garraina (*Garra* + *Garra*-like cyprinids). Our molecular results show that *Cophecheilus bamen* and *Prolixicheilus longisulcus* form a clade, which is the sister to all other members of the karst group.

**Figure 3.**
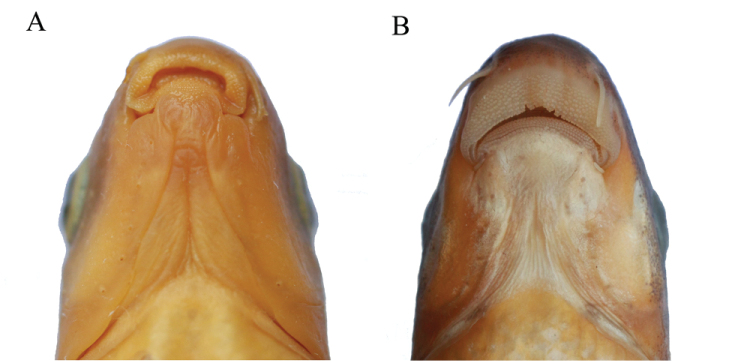
Ventral view of the mouth morphology. **A** *Stenorynchoacrum xijiangensis* **B** *Rectoris posehensis*.

Zheng et al. (2010) tried to elucidate the phylogenetic position of *Stenorynchoacrum*. Insufficient samples and relatively low node support resulted in an inconclusive phylogenetic position. Our results suggest that the species of *Rectoris* form a monophyletic group, and that *Stenorynchoacrum xijiangensis* forms the sister taxon to *Rectoris* with strong support. Although *Stenorynchoacrum* and *Rectoris* are genetically closely related, *Stenorynchoacrum* is morphologically distinct from the species of *Rectoris* by the following combination of characteristics: middle part of rostral cap undeveloped, narrow, only covering the base of the upper jaw, both sides of rostral cap well-developed and extending upward (vs. rostral cap developed, covering upper jaw completely); lower lip modified into fleshy pad (vs. lower lip not modified) (Fig. 3).

**Figure 4.**
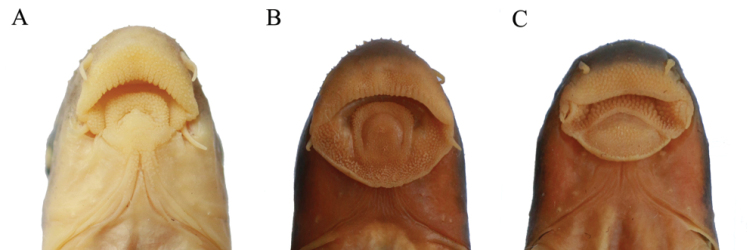
Ventral view of the mouth morphology. **A** *Hongshuia megalophthalmus* **B** *Discogobio brachyphysallidos* **C** *Discocheilus wuluoheensis*.

The validity of *Hongshuia* has been discussed by Zheng et al. (2010), and its independent generic position has been supported therein. However, its phylogenetic position was uncertain because of the relatively low node support. Our results strongly support that *Hongshuia* is closely related to *Discogobio* and *Discocheilus*. These three genera share a fleshy central pad on the lower lip, and they are genetically closely related (Fig. 4). *Paraqianlabeo striatus* forms the sister taxon to *Pseudogyrinocheilus prochilus*, and then forms a lineage together with *Sinocrossocheilus labiatus* Su, Yang & Cui, 2003. *Paraqianlabeo striatus* can be easily distinguished from *Pseudogyrinocheilus prochilus* by upper lip present (vs. absent), rudimentary sucker present (vs. absent), and mental grooves present (vs. absent). Species with disc or fleshy central pad on the lower lip (with the exception of *Pseudogyrinocheilus prochilus*) form clade IV in our molecular results.

**Figure 5.**
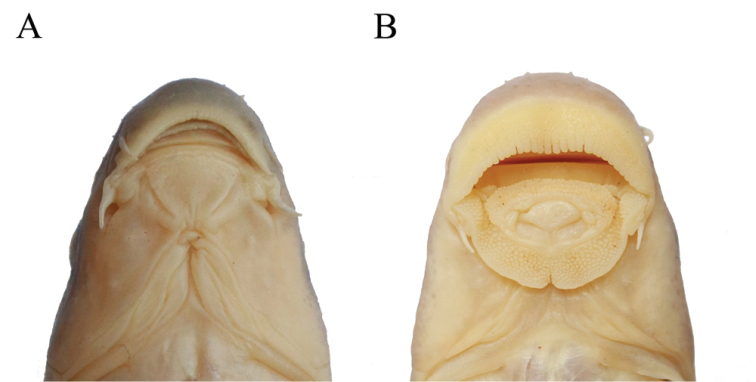
Ventral view of the mouth morphology. **A** *Sinigarra napoensis* **B** *Garra micropulvinus*.

In addition, Zhang and Zhou (2012) erected *Sinigarra* as a new genus because the authors considered the mental adhesive disc of *Sinigarra* more primitive compared to that of *Garra*, *Discogobio*, *Discocheilus* and *Placocheilus*. In fact, *Garra* is not a monophyletic group and the species allocated into *Garra* nowadays have been divided into several groups (Zheng et al. 2010; Yang et al. 2012). Due to the extensive distribution and complex mouth morphology, the taxonomy of *Garra* and its related genera is confused and awaits a comprehensive revision. *Sinigarra napoensis* Zhang & Zhou, 2012 shares the notch on the posterior margin of oral sucking disc with *Garra micropulvinus* Zhou, Pan & Kottelat, 2005. Our results showed that *Sinigarra napoensis* forms the sister taxon to *Garra micropulvinus*. The notch on the posterior margin of the oral sucking disc could be a homologous character for this group of fish (Fig. 5).

### Taxonomy of *Parasinilabeo*

*Parasinilabeo mutabilis* was described by Wu (1939) and was placed in the synonymy of *Parasinilabeo assimilis* Wu & Yao in Wu (1977). The genus *Parasinilabeo* has been a monotypic genus until 2000. Five new species, namely *Parasinilabeo longicorpus*, *Parasinilabeo maculatus* Zhang, 2000, *Parasinilabeo microps* Su, Yang & Cui, 2001, *Parasinilabeo longibarbus*, ﻿and *Parasinilabeo longiventralis*, have been successively described subsequently (Zhang 2000; Su et al. 2001; Zhu et al. 2006; Huang et al. 2007). The molecular results showed that the species of *Parasinilabeo* form a monophyletic lineage. In addition, *Parasinilabeo longicorpus* and *Parasinilabeo assimilis* form a lineage together. *Parasinilabeo longicorpus* was described as a new species because it was distinguished from *Parasinilabeo assimilis* by a more slender body (body depth 14.7–18.9 % of standard length vs. 23.3–26.3) and a lower caudal peduncle (caudal-peduncle depth 8.9–11.8 % of standard length vs. 12.1–14.1) (Zhang 2000). With the exception of the metric differences, there are not any other stable characters that can be used to effectively distinguish specimens. Moreover, the genetic distance of Cyt *b* gene between *Parasinilabeo assimilis* and *Parasinilabeo longicorpus* is 0.016, which is lower than the distance between *Parasinilabeo assimilis* and *Parasinilabeo longibarbus* (0.078) and that between *Parasinilabeo assimilis* and *Parasinilabeo longiventralis* (0.019). This low level of genetic variation is consistent with the morphological evidences. Therefore, *Parasinilabeo longicorpus* might be the synonym of *Parasinilabeo assimilis* and the comprehensive revision of this genus is needed.

### Taxonomy of *Ptychidio*

*Ptychidio macrops* Fang, 1981 was closely related to *Ptychidio jordani* Myers, 1930 in our results. *Ptychidio macrops* was distinguished from *Ptychidio jordani* by a larger eye (more than 25% of head length vs. less), shorter tassel (less than eye diameter vs. longer) and shorter rostral barbels (reaching anterior margin of eyes vs. reaching beyond). This situation is similar as that of *Parasinilabeo longicorpus* and *Parasinilabeo assimilis*. With the exception of the metric differences, there are not any other stable characters that can be used to effectively distinguish specimens. Moreover, the genetic distances of Cyt *b* gene between *Ptychidio jordani* and *Ptychidio macrops* is 0.011, which is lower than the distance between *Ptychidio jordani* and *Ptychidio longibarbus* Chen & Chen, 1989 (0.028). Similarly, in view of the close genetic relationship and the morphometric differences, *Ptychidio macrops* might be the synonym of *Ptychidio jordani* and the comprehensive revision of this genus is needed.

## Supplementary Material

XML Treatment for Prolixicheilus

## Supplementary materials

Supplementary material 1 Table S1 Data type: molecular data Explanation note: Taxa included in this study and accession numbers of sequences in GenBank. This dataset is made available under the Open Database License (http://opendatacommons.org/licenses/odbl/1.0/). The Open Database License (ODbL) is a license agreement intended to allow users to freely share, modify, and use this Dataset while maintaining this same freedom for others, provided that the original source and author(s) are credited.Lan-Ping Zheng, Xiao-Yong Chen, Jun-Xing Yang

Supplementary material 2 Figure S1 Data type: figure Explanation note: Scatter plots for the number of transitions and tranversions versus the F84 distance of all sequences. This dataset is made available under the Open Database License (http://opendatacommons.org/licenses/odbl/1.0/). The Open Database License (ODbL) is a license agreement intended to allow users to freely share, modify, and use this Dataset while maintaining this same freedom for others, provided that the original source and author(s) are credited.Lan-Ping Zheng, Xiao-Yong Chen, Jun-Xing Yang
