# Supplementary material for: Molecular systematics of the Labeonini inhabiting the karst regions in southwest China (Teleostei, Cypriniformes)
Source: Zookeys. 2016 Aug 23;(612):133–48. doi: 10.3897/zookeys.612.9085 (PMC5027668; doi:10.3897/zookeys.612.9085)
Supplement: Supplementary material 1 — Table S1 [file zookeys-612-133-s001.doc]

Table S1. Taxa included in this study and accession numbers of sequences in GenBank.

| [Subfamily](http://zipcodezoo.com/Key/Animalia/Cultrinae_Subfamily.asp) /tribe | Taxon | Voucher specimen | Sampling location | Accession No. | | | | | | |
| --- | --- | --- | --- | --- | --- | --- | --- | --- | --- | --- |
|  |  |  |  | Nuclear gene | | | | Mitochondrial gene | | |
|  |  |  |  | RAG1 | RH | EGR2B | IRBP | *Cyt* b | COI | 16S rRNA |
| [**Outgroups**](http://zipcodezoo.com/Key/Animalia/Cultrinae_Subfamily.asp) |  |  |  |  |  |  |  |  |  |  |
| Xenocypridinae |  |  |  |  |  |  |  |  |  |  |
|  | *Ischikauia steenackeri* |  |  | EU292687 | FJ197046 | EU409744 | FJ197096 | AB239601 | AB239601 | AB239601 |
|  | *Nipponocypris sieboldii* |  |  | EU292713 | FJ197069 | FJ531312 | FJ197120 | AB218898 | AB218898 | AB218898 |
|  | *Opsariichthys uncirostris* |  |  | FJ197126 | FJ197068 | FJ531300 | FJ197119 | AB218897 | AB218897 | AB218897 |
| [Cyprininae](http://zipcodezoo.com/Key/Animalia/Cultrinae_Subfamily.asp) |  |  |  |  |  |  |  |  |  |  |
| Barbini | *Barbus barbus* |  |  | EU711147 | FJ197049 |  | FJ197099 | AB238965 | AB238965 | AB238965 |
| Cyprinini | *Carassius auratus* |  |  | DQ196520 | L11863 |  | X80802 | AB006953 | AB006953 | AB006953 |
| Cyprinini | *Cyprinus carpio* |  |  | AY787040 | U02475 | GQ913553 | FJ197101 | AP009047 | AP009047 | AP009047 |
| Poropuntiini | *Barbonymus gonionotus* |  |  | EU711146 | FJ197048 | JX074509 | FJ197098 | AB238966 | AB238966 | AB238966 |
| Schizopygopsini | *Gymnocypris przewalskii* |  |  | EU711149 | FJ197051 | FJ531294 | FJ197102 | AB239595 | AB239595 | AB239595 |
| Smiliogastrini | *Enteromius trimaculatus* |  |  | EU711148 | FJ197050 | JX074507 | FJ197100 | AB239600 | AB239600 | AB239600 |
| Smiliogastrini | *Pethia ticto* |  |  | EU711152 | FJ197054 |  | FJ197105 | AB238969 | AB238969 | AB238969 |
| Spinibarbini | *Spinibarbus denticulatus* | KIZMR20080136 | Libo, Guizhou Prov. | GU086506 | GU086468 | KT633654 | KT633669 | GU086544 | GU086582 | GU168743 |
| [Labeonini](http://zipcodezoo.com/Key/Animalia/Cultrinae_Subfamily.asp) | *Bangana lippa* | KIZCXY2008077 | Xishuangbanna, Yunnan Prov. | GU086525 | GU086487 | JN160213 | JN160249 | GU086563 | GU086601 | GU168762 |
|  | *Bangana tonkinensis* | KIZ200401163 | Mojiang, Yunnan Prov. | GU086524 | GU086486 | JN160212 | JN160248 | GU086562 | GU086600 | GU168761 |
|  | *Labeo batesii* |  |  | EU711150 | FJ197052 | GQ913576 | FJ197103 | AB238967 | AB238967 | AB238967 |
|  | *Labeo senegalensis* |  |  | EU711151 | FJ197053 | GQ913583 | FJ197104 | AB238968 | AB238968 | AB238968 |
|  | *Labeo stolizkae* | KIZCXY20060059 | Ruili, Yunnan Prov. | GU086498 | GU086460 | JN160221 | JN160257 | GU086536 | GU086574 | GU168735 |
|  | *Garra imberba* | KIZWWY20080051 | Wenshan, Yunnan Prov. | GU086530 | GU086492 | JN160217 | JN160253 | GU086568 | GU086606 | GU168767 |
|  | *Garra micropulvinus* | KIZWWY20080244 | Wenshan, Yunnan Prov. | GU086529 | GU086491 | JN160216 | JN160252 | GU086567 | GU086605 | GU168766 |
|  | *Garra mirofrontis* | KIZ20050325008 | Yunxian, Yunnan Prov. | GU086527 | GU086489 | JN160215 | JN160251 | GU086565 | GU086603 | GU168764 |
|  | *Garra orientalis* | KIZ200406230 | Longlin, Guangxi Prov. | GU086526 | GU086488 | JN160214 | JN160250 | GU086564 | GU086602 | GU168763 |
|  | *Garra tengchongensis* | KIZCXY20060137 | Tengchong, Yunnan Prov. | GU086528 | GU086490 | N/A | N/A | GU086566 | GU086604 | GU168765 |
|  | *Gonorhynchus bicornis* | KIZ05207 | Lushui, Yunnan Prov. | GU086514 | GU086476 | JN160236 | JN160272 | GU086552 | GU086590 | GU168751 |
|  | *Gonorhynchus burmanicus* | KIZCXY20060030 | Longchuan, Yunnan Prov. | GU086497 | GU086459 | JN160220 | JN160256 | GU086535 | GU086573 | GU168734 |
|  | *Placocheilus cryptonemus* | KIZ05169 | Lushui, Yunnan Prov. | GU086494 | GU086456 | JN160218 | JN160254 | GU086532 | GU086570 | GU168731 |
|  | *Sinigarra napoensis* | KIZZLP20130171 | Jingxi, Guangxi Prov. | KT633697 | KT633712 | N/A | KT633682 | KT633651 | KT633636 | KT633621 |
| [**Ingroups**](http://zipcodezoo.com/Key/Animalia/Cultrinae_Subfamily.asp) |  |  |  |  |  |  |  |  |  |  |
|  | *Cophecheilus bamen* | KIZDLN20080002 | Jingxi, Guangxi Prov. | KT633685 | KT633700 | KT633655 | KT633670 | KT633639 | KT633624 | KT633609 |
|  | *Discocheilus wuluoheensis* | KIZMR20080429 | Luoping, Yunnan Prov. | GU086531 | GU086493 | JN160211 | JN160247 | GU086569 | GU086607 | GU168768 |
|  | *Discocheilus wui* | KIZDLN20080045 | Leye, Guangxi Prov. | GU086523 | GU086485 | JN160209 | JN160245 | GU086561 | GU086599 | GU168760 |
|  | *Discogobio bismargaritus* | KIZYJ20100042 | Bama,Guangxi Prov. | KT633691 | KT633706 | KT633661 | KT633676 | KT633645 | KT633630 | KT633615 |
|  | *Discogobio brachyphysallidos* | KIZMR20080368 | Luoping, Yunnan Prov. | GU086509 | GU086471 | JN160232 | JN160268 | GU086547 | GU086585 | GU168746 |
|  | *Discogobio laticeps* | KIZYJ2013019 | Napo, Guangxi Prov. | KT633695 | KT633710 | KT633665 | KT633680 | KT633649 | KT633634 | KT633619 |
|  | *Discogobio longibarbatus* | KIZ201109 | Fuxianhu, Yunnan Prov. | KT633698 | KT633713 | KT633667 | KT633683 | KT633652 | KT633637 | KT633622 |
|  | *Discogobio macrophysallidos* | KIZMR20080335 | Luoping, Yunnan Prov. | GU086511 | GU086473 | JN160233 | JN160269 | GU086549 | GU086587 | GU168748 |
|  | *Discogobio multilineatus* | KIZYJ20100026 | Bama,Guangxi Prov. | KT633690 | KT633705 | KT633660 | KT633675 | KT633644 | KT633629 | KT633614 |
|  | *Discogobio tetrabarbatus* | KIZMR20080058 | Libo, Guizhou Prov. | GU086518 | GU086480 | JN160207 | JN160243 | GU086556 | GU086594 | GU168755 |
|  | *Discogobio yunnanensis* | KIZ2006003377 | Jinning, Yunnan Prov. | GU086507 | GU086469 | JN160230 | JN160266 | GU086545 | GU086583 | GU168744 |
|  | *Hongshuia megalophthalmus* | KIZMR20080234 | Tian'e, Guangxi Prov. | GU086517 | GU086479 | JN160204 | JN160240 | GU086555 | GU086593 | GU168754 |
|  | *Hongshuia microstomatus* | KIZMR200800576 | Libo, Guizhou Prov. | GU086510 | GU086472 | N/A | N/A | GU086548 | GU086586 | GU168747 |
|  | *Hongshuia paoli* | KIZDLN20080003 | Lingyun, Guangxi Prov. | GU086522 | GU086484 | JN160238 | JN160274 | GU086560 | GU086598 | GU168759 |
|  | *Parasinilabeo assimilis* | KIZ20050625013 | Libo, Guizhou Prov. | GU086515 | GU086477 | JN160237 | JN160273 | GU086553 | GU086591 | GU168752 |
|  | *Parasinilabeo longibarbus* | KIZYJ20100005 | Fuchuan, Guangxi Prov. | KT633689 | KT633704 | KT633659 | KT633674 | KT633643 | KT633628 | KT633613 |
|  | *Parasinilabeo longicorpus* | KIZYJ20100056 | Du'an, Guangxi Prov. | KT633692 | KT633707 | KT633662 | KT633677 | KT633646 | KT633631 | KT633616 |
|  | *Parasinilabeo longicorpus* | KIZYJ20100004 | Guilin, Guangxi Prov. | KT633688 | KT633703 | KT633658 | KT633673 | KT633642 | KT633627 | KT633612 |
|  | *Parasinilabeo longiventralis* | KIZYJ2013033 | Fuchuan, Guangxi Prov. | KT633696 | KT633711 | KT633666 | KT633681 | KT633650 | KT633635 | KT633620 |
|  | *Pseudocrossocheilus bamaensis* | KIZMR20080141 | Du'an, Guangxi Prov. | GU086516 | GU086478 | JN160203 | JN160239 | GU086554 | GU086592 | GU168753 |
|  | *Pseudocrossocheilus liuchengensis* | KIZMR20080173 | Du'an, Guangxi Prov. | GU086502 | GU086464 | JN160225 | JN160261 | GU086540 | GU086578 | GU168739 |
|  | *Pseudocrossocheilus longibullus* | KIZ20050625001 | Libo, Guizhou Prov. | GU086495 | GU086457 | JN160210 | JN160246 | GU086533 | GU086571 | GU168732 |
|  | *Pseudocrossocheilus nigrovittatus* | KIZMR20080051 | Libo, Guizhou Prov. | GU086503 | GU086465 | JN160226 | JN160262 | GU086541 | GU086579 | GU168740 |
|  | *Pseudocrossocheilus papillolabrus* | KIZMR20080514 | Zhenfeng, Guizhou Prov. | GU086513 | GU086475 | JN160235 | JN160271 | GU086551 | GU086589 | GU168750 |
|  | *Pseudocrossocheilus tridentis* | KIZMR20080365 | Luoping, Yunnan Prov. | GU086508 | GU086470 | JN160231 | JN160267 | GU086546 | GU086584 | GU168745 |
|  | *Pseudogyrinocheilus prochilus* | KIZ200405021 | Yiliang, Yunnan Prov. | GU086496 | GU086458 | JN160219 | JN160255 | GU086534 | GU086572 | GU168733 |
|  | *Prolixicheilus* (*Pseudogyrinocheilus*) *longisulcus* | KIZDLN20080151 | Jingxi, Guangxi Prov. | KT633686 | KT633701 | KT633656 | KT633671 | KT633640 | KT633625 | KT633610 |
|  | *Ptychidio jordani* | KIZMR20080087 | Libo, Guizhou Prov. | GU086504 | GU086466 | JN160227 | JN160263 | GU086542 | GU086580 | GU168741 |
|  | *Ptychidio longibarbus* | KIZYJ20100067 | Du'an, Guangxi Prov. | KT633693 | KT633708 | KT633663 | KT633678 | KT633647 | KT633632 | KT633617 |
|  | *Ptychidio macrops* | KIZYJ20100003 | Pingxiang, Guangxi Prov. | KT633687 | KT633702 | KT633657 | KT633672 | KT633641 | KT633626 | KT633611 |
|  | *Paraqianlabeo striatus* | KIZMR20080505 | Zunyi, Guizhou Prov. | GU086512 | GU086474 | JN160234 | JN160270 | GU086550 | GU086588 | GU168749 |
|  | *Rectoris luxiensis* | KIZZLP20110313 | Huaihua, Hunan Prov. | KT633699 | KT633714 | KT633668 | KT633684 | KT633653 | KT633638 | KT633623 |
|  | *Rectoris posehensis* | KIZMR20080065 | Libo, Guizhou Prov. | GU086519 | GU086481 | JN160205 | JN160241 | GU086557 | GU086595 | GU168756 |
|  | *Semilabeo notabilis* | KIZYJ20100069 | Du'an, Guangxi Prov. | KT633694 | KT633709 | KT633664 | KT633679 | KT633648 | KT633633 | KT633618 |
|  | *Semilabeo obscurus* | KIZMR20080085 | Libo, Guizhou Prov. | GU086505 | GU086467 | JN160228 | JN160264 | GU086543 | GU086581 | GU168742 |
|  | *Sinocrossocheilus labiatus* | KIZZLP20090021 | Tongzi, Guizhou Prov. | GU086520 | GU086482 | JN160206 | JN160242 | GU086558 | GU086596 | GU168757 |
|  | *Stenorynchoacrum xijiangensis* | KIZDLN20080001 | Guilin, Guangxi Prov. | GU086521 | GU086483 | JN160208 | JN160244 | GU086559 | GU086597 | GU168758 |
